# Implementing monitoring triggers and matching of triggered and control sites in the TEMPER study: a description and evaluation of a triggered monitoring management system

**DOI:** 10.1186/s13063-019-3301-z

**Published:** 2019-04-17

**Authors:** Carlos Diaz-Montana, William J. Cragg, Rahela Choudhury, Nicola Joffe, Matthew R. Sydes, Sally P. Stenning

**Affiliations:** 1 MRC Clinical Trials Unit at UCL, Institute of Clinical Trials & Methodology, University College London, 90 High Holborn 2nd Floor, London, WC1V 6LJ UK; 2 Clinical Trials Research Unit, Leeds Institute of Clinical Trials Research, University of Leeds, Leeds, UK

**Keywords:** Triggered monitoring, Risk-based monitoring, Data management system

## Abstract

**Background:**

Triggered monitoring in clinical trials is a risk-based monitoring approach where triggers (centrally monitored, predefined key risk and performance indicators) drive the extent, timing, and frequency of monitoring visits. The TEMPER study used a prospective, matched-pair design to evaluate the use of a triggered monitoring strategy, comparing findings from triggered monitoring visits with those from matched control sites. To facilitate this study, we developed a bespoke risk-based monitoring system: the TEMPER Management System.

**Methods:**

The TEMPER Management System comprises a web application (the front end), an SQL server database (the back end) to store the data generated for TEMPER, and a reporting function to aid users in study processes such as the selection of triggered sites. Triggers based on current practice were specified for three clinical trials and were implemented in the system. Trigger data were generated in the system using data extracted from the trial databases to inform the selection of triggered sites to visit. Matching of the chosen triggered sites with untriggered control sites was also performed in the system, while data entry screens facilitated the collection and management of the data from findings gathered at monitoring visits.

**Results:**

There were 38 triggers specified for the participating trials. Using these, 42 triggered sites were chosen and matched with control sites. Monitoring visits were carried out to all sites, and visit findings were entered into the TEMPER Management System. Finally, data extracted from the system were used for analysis.

**Conclusions:**

The TEMPER Management System made possible the completion of the TEMPER study. It implemented an approach of standardising the automation of current-practice triggers, and the generation of trigger data to inform the selection of triggered sites to visit. It also implemented a matching algorithm informing the selection of matched control sites. We hope that by publishing this paper it encourages other trialists to share their approaches to, and experiences of, triggered monitoring and other risk-based monitoring systems.

## Background

Risk-based monitoring (RBM) strategies are increasingly advocated in clinical trials, with the aim of reducing monitoring costs while maintaining or improving data quality and integrity and participant protection [1–5]. The approach is also encouraged by regulators; the International Conference of Harmonisation (ICH) Good Clinical Practice (GCP) guidance (E6[R2]) advises trialists to “develop a systematic, prioritised, risk-based approach to monitoring clinical trials” [6]. RBM tools support one or both of two components of RBM: an initial risk assessment, which determines the overarching monitoring strategy, and support for ongoing monitoring activities in response to the risks identified [7, 8], including determining the nature and frequency of on-site monitoring visits.

Conventional approaches to on-site monitoring tend to be conservative, involving routine, often frequent [9], visits to each site. The frequency may be based only on the initial risk assessment. Triggered monitoring (or targeted monitoring) is an RBM approach in which the extent, timing, and frequency of monitoring visits are driven by centrally monitored triggers. These can be described as predefined, trial-specific key risk and performance indicators that fire when the metric they observe crosses a pre-set acceptability threshold. Triggers may be quantitative measurements calculated using centrally held trial data, or subjective assessments, and are reviewed regularly to prioritise sites for visits. Examples of metrics include recruitment levels, data return rates, missing data levels, incidence of protocol deviations, and safety reporting timelines.

The Targeted Monitoring: Prospective Evaluation and Refinement (TEMPER) study [10] used a prospective, matched-pair design to evaluate the use of a triggered site monitoring strategy. It compared findings from triggered monitoring visits with those from matched control sites that were not prioritised for visiting at that time, to determine if the strategy was effective at distinguishing sites with a higher risk of concerning, previously unknown, monitoring findings from those at lower risk. Three multi-centre cancer trials at the Medical Research Council Clinical Trials Unit (MRC CTU) at University College London (UCL), with 156 UK sites in total, participated in TEMPER.

To allow the evaluation of this triggered monitoring strategy for the study, we developed the TEMPER Management System (TEMPER-MS), an RBM tool (computer software) to systematically define triggers and summarise their status.

The results of the TEMPER study have been reported by Stenning et al. [10] and further details of the study conduct and included trials are therein explained. This paper aims to describe the main procedures and overall design of TEMPER-MS, evaluate its functioning and potential for further development, and inform trialists wishing to implement similar RBM tools. For ease of reference, this paper contains some details that were previously reported in [10] including the description of the matching algorithm and part of Table 2.

## Methods

We required a system that allowed: 1) generation of trigger data (to evaluate the triggers) using data held in the participating trial databases; 2) selection of triggered sites based on the trigger data; 3) pairing of the chosen triggered sites with control sites based on specified similarity criteria; and 4) collection and management of data from findings gathered at all the monitoring visits.

TEMPER-MS is a bespoke software system developed in-house at MRC CTU by the author (following the unit’s standard procedures). It comprises a web application (the front end) developed in ASP.NET web forms, an SQL server database (the back end) which stored the data generated for TEMPER, and reports developed in SQL server reporting services, made available to aid users in study processes such as the selection of triggered sites. The system also included data entry screens for collecting monitoring visit data. Developing a bespoke system was regarded as the best option to meet all study requirements, some of which (including the matching process) were very particular to TEMPER. There was also the expertise available at MRC CTU to develop a validated computer system to meet these requirements.

### System flow overview

**Fig. 1:**
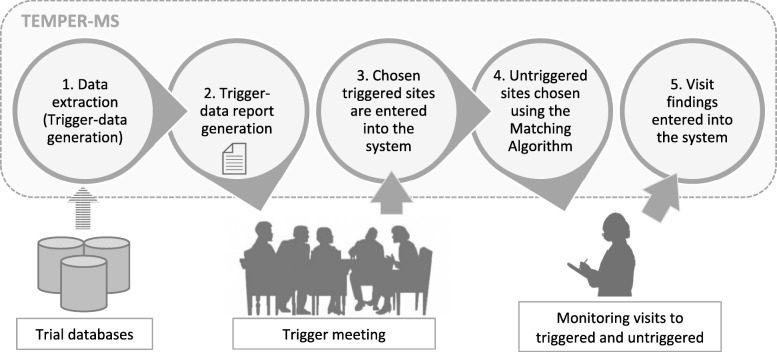
TEMPER Management System (TEMPER-MS) main functions and their interaction with external data and processes

Figure 1 shows the main functions of TEMPER-MS and how external processes, such as the trigger meetings and monitoring visits, were aided by the system.

Trial teams held 3- to 6-monthly trigger meetings with the TEMPER team to choose triggered sites for monitoring. This frequency reflected typical practice by the trial teams according to the stage of the trial (e.g. in recruitment or follow-up). A data extraction process was run in TEMPER-MS before each meeting which involved data retrieval from the trial database, aggregation per site, and further processing to produce trigger data. After extraction, a trigger data report was generated and used in the trigger meeting to guide the prioritisation of triggered sites.

For each of the chosen triggered sites an untriggered site was matched as a control site with the help of the TEMPER-MS matching algorithm. Each site pair was visited, and the monitoring findings were entered into the system.

### Trigger development

For each participating trial, a list of triggers was specified by the trial team. Each trigger specification began with a plain English description (narrative) explaining the conditions under which it should fire. The majority of narratives were refinements of criteria already in use by trial teams, with the trials being ongoing when TEMPER started.

Most narratives were implemented as automatic triggers in TEMPER-MS, i.e. the triggers were automatically evaluated using data extracted from the trial databases. To enable a consistent implementation of automatic triggers into the system, each narrative was formatted into a standard inequality rule. This is the relationship between a given trigger threshold and the quotient of a metric Sample over a Population:

$$ \frac{Sample}{Population}<> Threshold $$

where the Population is the relevant total number of assessments of the observed metric, the Sample (generally a subset of the Population) is a sample of the metric, and the inequality symbol ‘<>’ denotes either ‘<’, ‘≤’, ‘>’, or ‘≥’.

**Fig. 2:**
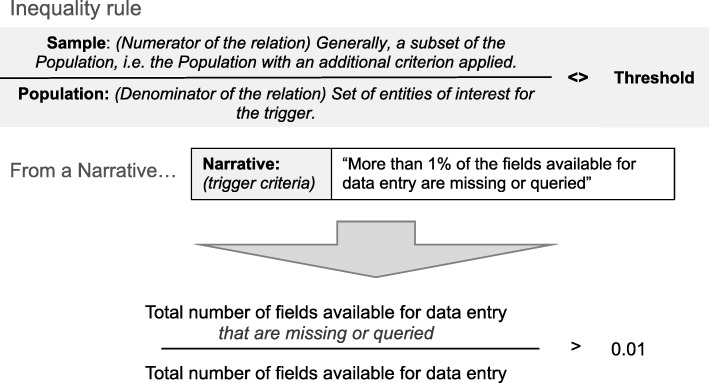
Example of a narrative formatted into an inequality rule for an automatic trigger

Figure 2 shows an example narrative (‘More than 1% of the fields available for data entry are missing or queried’) expressed as an inequality rule.

In some instances, the Population was a fixed value. For example, a recruitment trigger might have Sample = “total number of patients registered at a site” and Population = “the recruitment target set for the trial”. A trigger could be set to fire if a site had already passed a percentage (threshold) of the overall recruitment target.

For automatic triggers, the Population and Sample were calculated by TEMPER-MS using data extracted from the trial database, and were subsequently stored in the TEMPER-MS database with the user-defined threshold. The data extraction processes for each automatic trigger were tested by the corresponding trial team, verifying that the data generated accurately summarised the data of interest in the trial database. After the Population and Sample were obtained, the inequality rule was evaluated as either ‘true’ or ‘false’ (i.e. is the rule met?). Automatic triggers sometimes had pre-conditions in their narrative that needed to be met for trigger data to be generated; for instance, an inequality rule might be evaluated only if there were a minimum number of registered patients at the site.

When data were not available in the trial database to implement an automatic trigger, manual triggers were created in the system allowing users to set their firing status manually when the conditions in their narratives were met. Manual triggers did not require an inequality rule. Examples include triggers using data from external sources (e.g. protocol deviation logs held outside the trial database), and triggers based on subjective interpretation (e.g. concerns about site conduct identified by trial team members).

### Fine tuning triggers

Each trigger had an associated weight (default = 1) specifying its importance relative to other triggers. In some cases, it could also be used to define for-information-only triggers to highlight features of the trial conduct of certain sites, but where their occurrence would not be included as part of the assessment to choose triggered sites. For these cases, a value of zero (0) was assigned to the trigger weight.

In the trigger data generation, a score was calculated for every trigger–site combination using the trigger’s weight as follows:

IF trigger fires for the site, THEN score = weight, OTHERWISE score = 0.

After the trigger’s scores were calculated, a site score was obtained for each site as the summation of all scores associated with the site. The trigger data report generated for the trigger meeting listed sites sorted by their site score.

Some triggers were designed to fire only when their rule was met at consecutive trigger meetings (i.e. it would be necessary that the inequality rule was ‘true’ two or more times in a row for the trigger to fire). This could be used to distinguish sites that were not improving over time from those with temporary problems. To include this behaviour in the system, a real number between zero and one, called frequency, was associated with each trigger. Every time a trigger rule was ‘true’, the frequency was added to a stored cumulative variable, and if the result of this addition was greater than or equal to one (> = 1) the trigger would fire. The stored cumulative variable was reset to zero if the rule was ‘false’. The majority of triggers had a frequency = 1 (i.e. the trigger fired every time the trigger rule was met). Some triggers had a frequency of 0.5, meaning their rule had to be met twice in a row in order to fire.

### Matching algorithm: obtaining untriggered matched sites

Untriggered sites had to meet the following criteria: 1) not previously visited as an untriggered site; 2) site in the UK (i.e. only UK triggered sites were selected); 3) site score was less than the triggered site’s score and, if non-zero, low enough that the trial team would not be considering visiting at this time; and 4) site was ‘similar’ to the triggered site in terms of the number of patients randomised and time since first patient randomised. These ‘matching’ factors were chosen through discussion by the study development team.

**Fig. 3:**
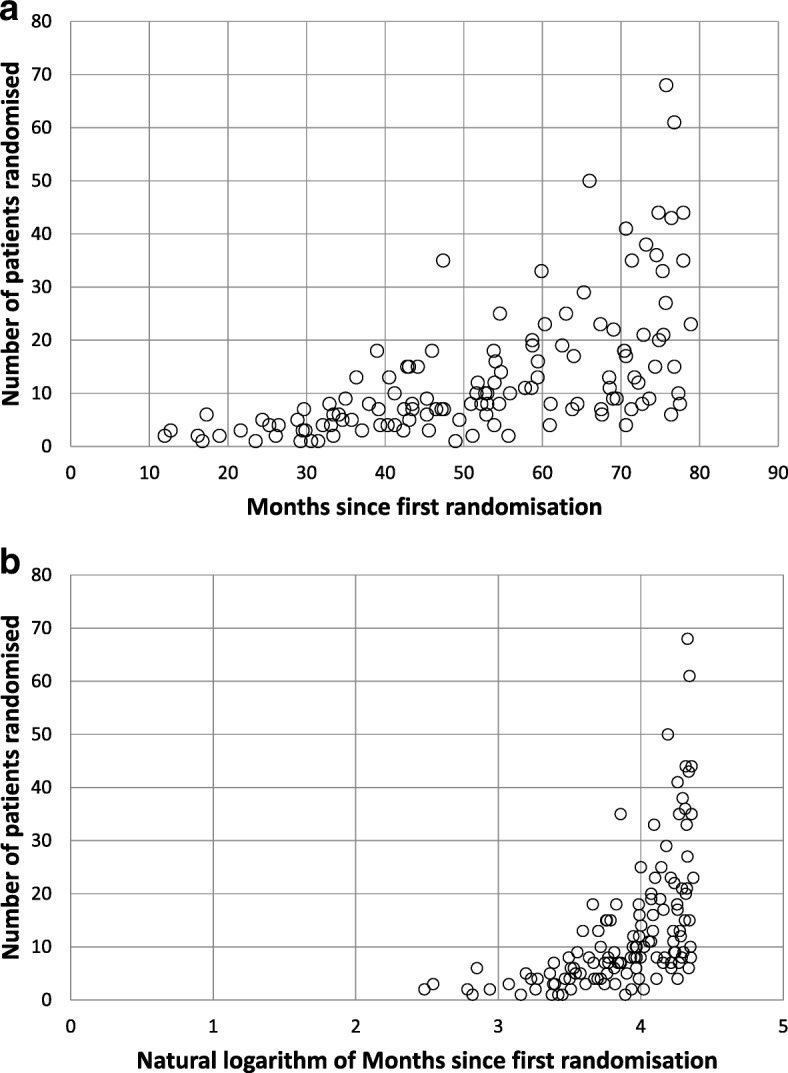
Graphical representation of the similarity of sites of a participant trial. Data are from a particular point in time (20 February 2014). Sites are plotted according to two variables: number of patients randomised and time since first site randomisation. The latter variable is shown in the *x* axis as **a** number of months and **b** natural logarithm of the number of months

We can visualize the two similarity variables in the scatterplots shown in Fig. 3, where a snapshot of site data from a participating trial is used for illustrative purposes. The similarity of two sites can be viewed as how close they are on these graphs. Figure 3a shows the number of months since the first site randomisation on the *x* axis, while Fig. 3b shows the natural logarithm of the number of months. The natural logarithm was used in TEMPER-MS to adjust the time since first randomisation variable because, for instance, a 12-month difference in recruitment time was seen as more meaningful between sites starting 3 and 15 months ago than between sites starting 4 and 5 years ago.

We can preliminarily define a matching score between two sites, where lower scores mean sites are more similar, as the (Euclidean) distance between their data points in this bi-dimensional space:

$$ distance=\sqrt{{\left({x}_2-{x}_1\right)}^2+{\left({y}_2-{y}_1\right)}^2} $$

where (x_2_ – x_1_) is the difference between the natural logarithms of the months since first randomisation of the two sites, and (y_2_ – y_1_) is the difference between the two sites regarding the number of patients.

Although potential untriggered sites did not have to have a zero site score, to be considered by the trial team as an untriggered site their score had to be low. In order to prioritise sites with lower scores in the untriggered site selection (i.e. to penalise sites with higher scores), a penalty was added to the distance to complete the matching score definition:

$$ matching\ score= distance+ penalty $$

$$ penalty=\left(\left[ site\ score\right]\ast p\right) $$

By increasing the matching score value of the candidate site, the penalty decreased its eligibility as an untriggered site proportionally to the site’s score. A penalty factor ‘*p*’ (a proportionality constant) was introduced to determine the weight of the site score in the final matching score calculation. The optimal value of *p* would not necessarily be the same for each trial since the number of triggers assessed, and the frequency with which each trigger fired, varied across trials. The value of *p* for each participating trial was determined by the TEMPER statistician (SPS), based on testing a range of values of *p* for each trial and making a subjective assessment of the adequacy of the matches selected in terms of the matching factors, the matched site score, and the difference in site scores within the pairs. While *p* was chosen in a subjective manner, it was then fixed at the end of testing and applied consistently to all selections in the live study; it could not therefore be used to manipulate matched site selection.

Once the triggered sites were chosen and entered into TEMPER-MS, the matching algorithm was able to rank eligible untriggered matches according to their matching score. The highest ranked candidate (with lowest matching score) was selected by default as the untriggered match; exceptions are described in Stenning et al. [10].

## Results

### Trigger design

There were 38 triggers specified for the three participating trials, 31 of them automatic and 7 manual. Table 1 shows the triggers along with their category and an abridged narrative. Out of the 31 automatic triggers, three triggers were added to one of the trials (Trial 2, triggers 11, 12, and 13 in Table 1) after the TEMPER study had started following a trigger meeting where it was agreed that the additional triggers would be useful. Thresholds were also adjusted for three automatic triggers during the project (Trial 1, trigger 5; and Trial 2, triggers 3 and 5). One of the seven manual triggers was added after the study had started (Trial 3, trigger M2 in Table 1).

**Table 1:** List of automatic and manual triggers with categories and abridged narratives

Trigger ID	Type^a^	Category	Abridged narrative	Firing rate^b^
Trial 1				
1	Au	Protocol deviation (treatment)	Drug dose greater than expected dose	77%
2	Au	Data query rate (specific question)	Number of missing values of a specific field greater than a given percentage of the total of corresponding forms	2%
3	Au	SAE rate (high)	Number of patients with SAEs is greater than expected	0%
4	Au	High recruitment	Site has recruited more patients than a set target	11%
5	Au	Data query rate (overall)	Number of queried fields greater than a given percentage of the total number of available fields	4%
6	Au	Data query resolution time	Queried fields outstanding for more than a specified time greater than a given percentage of the total of queried fields	26%
7	Au	Overall CRF return rate	CRF return rate less than a given value	16%
8	Au	Protocol deviation (eligibility)	Enrolment of ineligible patient (date of surgery)	8%
9	Au	Protocol deviation (eligibility)	Enrolment of ineligible patient (date of bloods)	22%
10	Au	Protocol deviation (eligibility)	Enrolment of ineligible patient (date of scan)	24%
M1	Mn	General concern	General concern from protocol deviation log	2%
Trial 2				
1	Au	SAE rate (low)	Number of patients with SAEs is less than expected	4%
2	Au	High recruitment	Site has recruited more patients than a set target	0%
3	Au	Data query rate (overall)	Number of queried fields greater than a given percentage of the total number of available fields	7%
4	Au	Data query resolution time	Queried fields outstanding for more than a specified time greater than a given percentage of the total of queried fields	31%
5	Au	Overall CRF return rate	CRF return rate less than a given value	4%
6	Au	Protocol deviation (treatment)	Drug (1) dose greater than expected dose	0%
7	Au	Protocol deviation (treatment)	Drug (2) dose greater than expected dose	1%
8	Au	Protocol deviation (treatment)	Drug (3) dose greater than expected dose	0%
9	Au	Protocol deviation (treatment)	Drug (4) dose greater than expected dose	0%
10	Au	Protocol deviation (treatment)	Drug (4) given when data indicate it should have been withheld	43%
11	Au	Protocol deviation (treatment)	Drug (5) dose greater than expected dose	0%
12	Au	Protocol deviation (withdrawal rate)	Number of withdrawn patients more than a given percentage of the total number of patients	3%
13	Au	Protocol deviation (treatment)	Drug not given at correct dose	0%
M1	Mn	General concern	General concern following Trial Management Group meetings	2%
M2	Mn	Protocol deviation (procedure)	Important safety test missed	0%
M3	Mn	Protocol deviation (treatment)	Medication is not administered when a particular test result is below a specified value	0%
M4	Mn	Protocol deviation (treatment)	Drug (3) not given at correct dose in feasibility study centres	0%
Trial 3				
1	Au	Return rate, specific CRF	If death reported, has the relevant form been sent?	2%
2	Au	Return rate, specific CRF	If progression reported, has the relevant progression CRF been sent?	43%
3	Au	Overall CRF return rate	CRF return rate less than a given value	55%
4	Au	Return rate, specific CRF	More than a specified number of patients with last form received more than a given time	5%
5	Au	Data query rate (overall)	Number of queried fields greater than a given percentage of the total of available fields	2%
6	Au	Data query resolution time	Queried fields outstanding for more than a specified time greater than a given percentage of the total of queried fields	47%
7	Au	SAE rate (low)	Number of patients with SAEs is less than expected	18%
8	Au	SAE rate (high)	Number of patients with SAEs is greater than expected	3%
M1	Mn	Return rate, patient consent form	Consent forms return rate less than a given value	23%
M2	Mn	General concern	General concern following Trial Management Team review	1%

*CRF:* case report form, *SAE:* serious adverse event

^a^Type of trigger: Au: automatic; Mn: manual

^b^Firing rate: proportion of assessments in which the trigger was fired

**Fig. 4:**
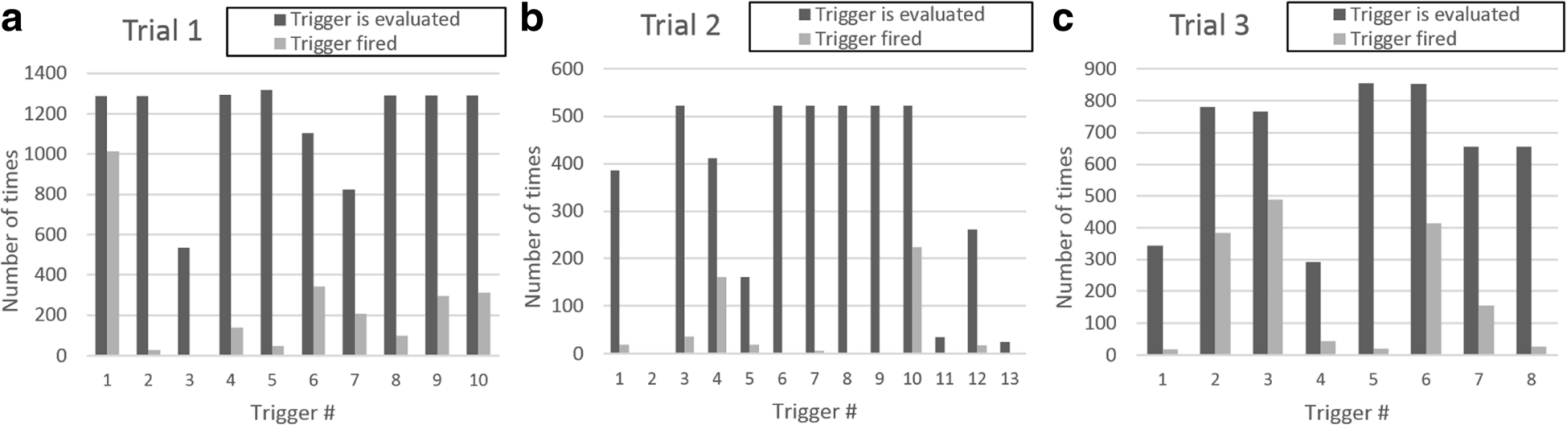
Comparison between times automatic triggers were evaluated versus times they fired for each participating trial. **a** Trial 1 (132 sites) held 10 trigger meetings; **b** Trial 2 (87 sites) held 6 meetings; **c** Trial 3 (127 sites) held 7 meeting

Figure 4 shows the number of times the automatic triggers were evaluated and trigger data were generated (the total number of sites at which the trigger was evaluated for all trigger meetings) versus the times they fired for each participating trial. The 31 automatic triggers fired 4525 times out of 21,126 times they were evaluated (21%), ranging from 0% (never firing) to 79% for individual triggers. The firing rate is affected by the thresholds set (for ordinal measures) as well as data quality. Manual triggers were set to fire 255 times across the three trials. During the course of TEMPER (19 April 2013 to 13 November 2015), triggers were evaluated before each planned trigger meeting and additionally as required to find matches for a site chosen for a triggered visit between planned meetings (as might occur if, for example, a serious protocol or GCP breach was identified). Recruitment to the trials started before, and continued after, these dates.

### Site selection and matching

**Fig. 5:**
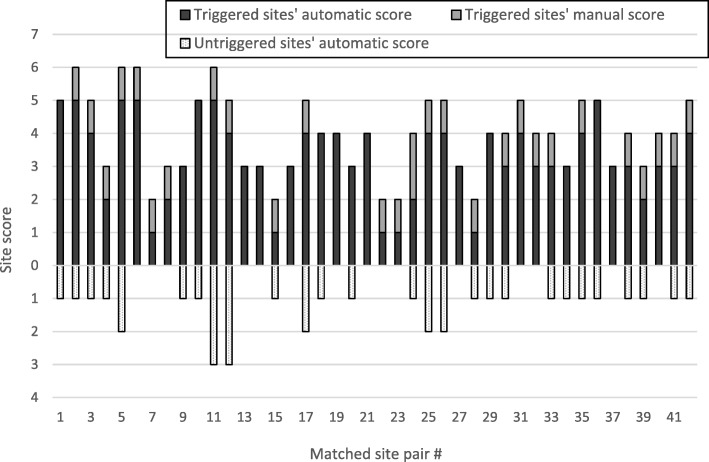
Site scores for triggered sites and their corresponding matched untriggered site. Triggered site scores show which part are due to automatic triggers versus manual triggers

There were 23 trigger meetings held where 42 triggered sites were chosen with the help of the sites’ scores calculated from the trigger data. The per-meeting median of number of sites chosen and paired with an untriggered site was 1.83; the number of triggered sites chosen at a given meeting was predominantly guided by the absolute site scores, but also took account of the trial team resources. Figure 5 shows the scores of the 42 site pairs. The score for the triggered sites from automatic triggers (83%) is distinguished from the score from manual triggers (17%). All the untriggered sites scores were due to automatic triggers (i.e. none of these sites had had manual triggers added).

The mean score of the triggered sites was 4.0 (range 2–6), the mean score of the untriggered sites was 0.8 (range 0–3), and the mean of the within-pair site score difference was 3.1 (range 1–6). The mean of the within-pair difference in number of patients was +8.5 and time since first randomisation was −1.4 months. Table 2 shows the maximum, mean, and minimum values of number of patients, time since first randomisation (number of months and natural logarithm of number of months), and score for triggered and untriggered sites, as well as the within-pair difference.

**Table 2:** Statistics for triggered and untriggered sites (number of patients, time since first randomisation, and score)

	Triggered sites				Untriggered sites				Within-pair difference			
	Patients^a^	Months^b^	Ln of months^c^	Score	Patients^a^	Months^b^	Ln of months^c^	Score	Patients^a^	Months^b^	Ln of months^c^	Score
Max	250	117.6	4.77	6	149	110.9	4.71	3	128	24.3	0.39	6
Mean	49.9	70.3	4.21	4.0	41.4	71.7	4.24	0.8	8.5	−1.4	−0.03	3.1
Min	3	30.6	3.42	2	3	34.6	3.54	0	−8	−39.3	−0.64	1

^a^Number of patients randomised at site

^b^Number of months since first site randomisation

^c^Natural logarithm of number of months since first site randomisation

From 156 UK sites participating in at least one of the three trials, 67 different sites (43%) were visited at least once during the course of TEMPER as triggered or untriggered sites.

## Discussion

The monitoring triggers and matching algorithm implemented in TEMPER-MS were key components of the TEMPER study. The system also allowed collection and management of monitoring findings for subsequent data analysis. This facilitated the primary analysis of the triggered monitoring strategy and further analysis of the individual triggers and their association with on-site monitoring findings.

### Trigger evaluation

The triggers used in the study were based on ‘current practice’ rather than being evidence-based; the TEMPER study aimed to test them empirically. The study showed that the triggers used did not discriminate as well as anticipated [10]. However, secondary analyses suggested that the current processes are able to identify sites at higher risk of critical on-site findings, and of major or critical findings relating to issues other than informed consent [10]. This suggests further refinement of the triggers may be warranted (see [10] for further discussion of this point). There remains a potential benefit in designing triggers based on existing organisational procedures and checks, which are a result of experience and expertise. Hurley et al. identified a lack of knowledge on how to define risks and translate them into monitoring activity as one of the main barriers for trialists to implementing RBM [8]; translating current practice into triggers could be a first step.

The system’s trigger data report, ranking sites by site score, sometimes highlighted sites that might otherwise have been overlooked. Similarly, with trigger metrics, trial teams are often more aware of some issues than others in their day-to-day work; for example, major protocol deviations may be more immediately obvious than a high data query rate.

Trigger scores informed rather than mandated the selection of triggered sites in the trigger meetings, which results in an important expert human component that reduces automation, while adding flexibility. The visual presentation in the data reports of the sites ranked by score allowed the team to decide how many sites to visit at that time, depending on the trigger scores, any additional external information on sites (such as staff turnover or concerns raised in other trials), and in part on the resources available. This also added to the flexibility of the model.

Our use of triggers included additional features such as frequency, used to highlight persistent trial conduct issues rather than one-off lapses, and weight, used to adjust the relative importance of each trigger in the final site score calculation. With further experience, the trial teams may have been able to quantify at least some of the human component referred to above by using the option to explicitly change the weighting of triggers over time (although available, this functionality was not used by any of the trial teams during the TEMPER study). It was possible to incorporate triggers for-information-only by setting their trigger weight to zero, thereby excluding them from the site score calculation but keeping them present in the data reports. An exploratory high recruitment trigger was used in two of the TEMPER trials to identify sites that have reached a fixed recruitment target, but it was not necessarily used in the selection of triggered sites to visit.

As with any triggered monitoring model, triggers in TEMPER-MS were mostly trial-specific and required tailored design and programming. Development of the automatic triggers required significant trial team resources and programming skills. However, the model described in this paper for standardising theoretical triggers into automated triggers by using an inequality rule is suitable for any potential data triggers. If adopted, it could help trialists to better understand triggers through the process of automating them, by identifying and discerning the inequality rule parts.

Initial triggers are, by definition, predefined; risks and areas of concern need to be identified, triggers programmed, and their initial threshold values set before monitoring begins. Nevertheless, triggers in TEMPER-MS also allowed customisation; thresholds, trigger weight (for score calculation), and frequency values could be fine-tuned as the trial progresses. New triggers could also be added in response to emerging risks.

### Binary versus multi-state triggers

Triggers in TEMPER were binary, which means either they fire (= 1) or they do not (= 0). In its simplest form, binary triggers fire if an observed metric crosses a single threshold. Binary triggers are more useful when the nature of the metric is also binary. For instance, if we want to evaluate safety concerns or protocol non-compliance, it is better to know if any safety breach or non-compliance event has occurred or not. In these cases, a trigger can be implemented with a Sample equal to the number of such events (i.e. Population = 1 and threshold set to 0), so the trigger fires if any event is recorded.

For other metrics, it may be more interesting to know the degree of an event occurring rather than if it has occurred at all. In these cases, a trigger output with more than two states could be more useful, such as a traffic light classification (green, amber, and red) for data return rates, or number of missing values. These multi-state triggers can be implemented by having a set of thresholds that classify the metric evaluation in the different states, which are represented by a real number between zero and one. The single threshold for binary triggers used in TEMPER will return just two states.

### Matching algorithm evaluation

The other important component of TEMPER-MS was the matching algorithm, which was designed to make possible the comparison of triggered sites with similar sites meeting fewer triggers, an aspect particular to the TEMPER study. The general idea of quantifying similarity between two entities (sites in this case) by using the Euclidean distance between the entities’ representations in a multi-dimensional plane according to the entities’ properties can be easily implemented in other models that need to quantify similarity. The addition of a penalty to the similarity equation provides the opportunity to deprioritise entities with a particular characteristic, if required.

### Future work

TEMPER-MS was the first system of its kind to be developed at MRC CTU, and is a reference point for future triggered monitoring systems and other RBM tools. The unit can build on the experiences gained from its design, development, and usage in future developments.

The inequality rule facilitated the integration of trigger data from different sources by proposing a simple way to standardise and aggregate extracted data, which was aimed to be easy to understand and implement. Other trialists can easily develop their own triggers in this model. The system retained each individual value calculated for every part of each rule, and their combination used in every trigger meeting. The availability of historic individual and aggregated data values of the trigger rules makes further analyses possible, such as the study of triggers over time to identify trends. The databases for each of the participating trials in TEMPER all used the same clinical data management system (Elsevier’s MACRO [11]). This facilitated the extraction and integration processes since the data structure at database table level was the same. However, this inequality rules model can potentially be applied to databases with different data structures.

We are looking to develop and test a comprehensive trigger management system, building on TEMPER-MS. This would include a central repository of triggers to facilitate the development of new, evolutionary triggers. Important general trends and patterns could also be identified across triggers, trials, and sites. The new trigger management system will incorporate data entry and management of visit findings to facilitate ongoing evaluation of triggers by explicitly linking those fired pre-visit to the severity and nature of on-site visit findings. This system could also incorporate putative triggers, the status of which would be recorded but not initially used to prioritise sites (by setting its weight to zero). These could then be analysed in conjunction with visit findings to look for evidence of their ability to predict on-site findings which, if successful, could be added to the new system alongside other emerging evidence-based triggers, while those triggers that do not appear to discriminate could be dropped.

Better trigger weights can be determined by group decision-making techniques, collaboratively by a cross functional team. The strategy described by Diani et al. [5] for deriving an overall risk score per site included a survey to determine the weights for each one of their risk factors. The survey was sent to their organisation’s members, asking them to rank the risk factors, previously also identified through a consensus exercise, “according to importance when assessing the need for intervening with an investigator site”. A percentage weight was then assigned to each risk factor based on the results of the survey.

Triggered monitoring can be complemented with other techniques such as Central Statistical Monitoring (CSM) as part of a wider monitoring strategy. CSM of key risk indicators uses statistical tests to analyse a large amount of data, identifying sites with abnormal patterns in specific data items [12] (which could be considered as additional triggers) or across all study data [2], potentially triggering an on-site visit. While CSM requires the volume of data to be reasonably large [2], making it unsuitable for small trials or delaying its application until enough data are available, triggered monitoring can be used in small data samples measuring single occurring events. Conversely, CSM may detect abnormalities that are missed by triggered monitoring, i.e. issues that are not concerning in isolation, but collectively indicate systemic trial conduct problems [2].

## Conclusion

The TEMPER-MS implemented an approach of standardising the automation of current-practice triggers, and provided the functionalities needed to generate trigger data and to present such data to inform the selection of triggered sites to visit. It also implemented a matching algorithm that incorporated concepts of similarity between sites and a penalty for poor-performing sites, informing the selection of matched control sites. By also including the facility to record the monitoring findings, it has allowed assessment of the discriminatory ability of the triggers used and helped highlight the need for improvement.

We encourage other trialists to share their approaches to, and experiences of, triggered monitoring. Implementation of similar systems in other trials will help evaluate alternative triggers and thresholds, in turn enhancing the evidence base around triggered monitoring approaches.

## Abbreviations

CSM: Central Statistical Monitoring
ICH: International Conference on Harmonisation
GCP: Good Clinical Practice
MRC CTU: Medical Research Council Clinical Trials Unit
RBM: Risk-based monitoring
TEMPER: Targeted Monitoring: Prospective Evaluation and Refinement
TEMPER-MS: TEMPER Management System
UCL: University College London
